# Dietary Quercetin Increases Colonic Microbial Diversity and Attenuates Colitis Severity in *Citrobacter rodentium*-Infected Mice

**DOI:** 10.3389/fmicb.2019.01092

**Published:** 2019-05-16

**Authors:** Rui Lin, Meiyu Piao, Yan Song

**Affiliations:** Department of Gastroenterology and Hepatology, General Hospital, Tianjin Medical University, Tianjin, China

**Keywords:** *Citrobacter rodentium*, colitis, gut microbiota, diet, quercetin, inflammatory bowel disease

## Abstract

Disturbed balance between microbiota, epithelial cells, and resident immune cells within the intestine contributes to inflammatory bowel disease (IBD) pathogenesis. The *Citrobacter rodentium*-induced colitis mouse model has been well documented. This model allows the analysis of host responses to enteric bacteria and facilitates improved understanding of the potential mechanisms of IBD pathogenesis. The current study evaluated the effects of dietary 30 mg/kg quercetin supplementation on *C. rodentium*-induced experimental colitis in C57BL/6 mice. Following dietary quercetin supplementation, the mice were infected with 5 × 10^8^ CFU *C. rodentium*, and the pathological effects of *C. rodentium* were measured. The results showed that quercetin alleviated the effects of *C. rodentium*-induced colitis, suppressed the production of pro-inflammatory cytokines, such as interleukin (IL)-17, tumor necrosis factor alpha, and IL-6 (*p* < 0.05), and promoted the production of IL-10 in the colon tissues (*p* < 0.05). Quercetin supplementation also enhanced the populations of *Bacteroides*, *Bifidobacterium*, *Lactobacillus,* and *Clostridia* and significantly reduced those of *Fusobacterium* and *Enterococcus* (*p* < 0.05). These findings indicate that dietary quercetin exerts therapeutic effects on *C. rodentium*-induced colitis, probably due to quercetin’s ability to suppress pro-inflammatory cytokines and/or modify gut microbiota. Thus, these results suggest that quercetin supplementation is effective in controlling *C. rodentium*-induced inflammation.

## Introduction

In the last decade, inflammatory bowel disease (IBD) has been one of the most frequently investigated human health issues associated with the gut microbiota (Kostic et al., 2014). More than 3.6 million people worldwide (Loftus, 2004) are affected with IBD, including Crohn’s disease (CD) and ulcerative colitis (UC), and the incidence of these diseases has been increasing in recent decades. The latter fact emphasizes the contribution of environmental factors to these diseases (Pillai, 2013). Thus, gut microbial communities have become a prominent research subject because of their effect on multiple aspects of health, such as IBD pathogenesis (Valdes et al., 2018).

The intestinal tract contains 10 trillion microorganisms (Dave et al., 2012) that are separated from the host’s mucosal immune cells by single layer of polarized epithelial cells play a crucial role in the development of the mucosal immune system. These symbiotic inhabitants, collectively known as the gut microbiota, also supply vital nutrients and limit the colonization of pathogenic microbes in the gut (Honda and Takeda, 2009). Evidence from both IBD patients and mouse models has shown that profound changes in the gut, such as intestinal microbiota development, play a major role in IBD pathogenesis (Gkouskou et al., 2014; Matsuoka and Kanai, 2015). Similar to enteropathogenic *Escherichia coli* (EPEC) and enterohemorrhagic *E. coli* (EHEC), *Citrobacter rodentium* is a member of the noninvasive group of attaching and effacing (A/E) bacteria that attach themselves to the intestinal epithelium and colonizes the host’s gut. At this point, the A/E pathogens induce alterations in the colonic tissue similar to those observed in cases of EPEC or EHEC infections in murine and human IBD (Law et al., 2013). A few models of infectious colitis exist, but in particular, the *C. rodentium*-induced colitis model (Law et al., 2013; Guan et al., 2016) has been well documented for studying the pathogenesis of host responses to enteric bacteria. This model can promote the understanding of the mechanism underlying IBD pathogenesis. Therefore, research related to *C. rodentium* is a key step for developing innovative prophylactic and therapeutic treatments.

Naturally found in fruits and vegetables, dietary antioxidant flavonoids are natural polyphenols. Recent studies have revealed that natural polyphenols exert potential preventative and therapeutic effects on various diseases (Ding et al., 2018; Hong and Piao, 2018). In certain organs, antioxidants provide inflammatory relief. Thus, natural polyphenols could be potential treatment options for IBD (Azuma et al., 2013). Quercetin is a flavonoid with antioxidant properties that is naturally present in most citrus fruits. It is considered to exert antidiabetic, antidepressant, and anti-inflammatory effects on cellular signaling pathways. In addition, quercetin inhibits tumor necrosis factor alpha (TNF-α) and interleukin (IL)-4 production in type I allergic reactions and decreases Th2-type cytokine production by basophils (Kim et al., 2014). It has also been proven to exert therapeutic effects on asthma, arthritis, and lung injury (Townsend and Emala, 2013); however, the precise mechanism by which it affects colitis is still unknown. Thus, this study was aimed at determining the potential effects of quercetin on *C. rodentium*-induced colitis in C57BL/6 mice.

## Materials and Methods

### *C. rodentium* Infection and Treatment/Animals and Experimental Design

This study was conducted according to the Chinese animal welfare guidelines after receiving approval from the Animal Care and Use Committee of General Hospital of the Tianjin Medical University. The study sample comprised pathogen-free female C57BL/6 mice that were kept under controlled conditions at 24 ± 2°C with a relative humidity of 60 ± 5% and a 12-h light/dark cycle (06:00–18:00). The control group (CTRL, *n* = 10) and the *C. rodentium*-infection group (CR-infection, *n* = 10) received a basal rodent diet. The quercetin group (QUE, *n* = 10) received a basal rodent diet supplemented with 30-mg/kg quercetin (Q0125, Sigma).

*C. rodentium* for infection was grown for 14 h in Luria Bertani (LB) broth containing 0.05-g/L nalidixic acid/mL. The cultures were then centrifuged at 3,000 × *g* for 10 min, and the pellets were resuspended in sterile phosphate-buffered saline (PBS). This *C. rodentium* culture with a final concentration of 5 × 10^8^ CFU/ml was used for infection. Briefly, mice were fed quercetin and/or basal rodent diet for 2 weeks as per the group allocation, and the mice in the CR-infection and QUE groups were infected with the 5 × 10^8^-CFU *C. rodentium* culture by gavage at 9:00 the next day. Subsequently, each mouse was housed in an individual cage to avoid reinfection from littermates. On day 7 post-infection, all mice were euthanized by CO_2_ asphyxiation.

The colonic mucosal tissues of all mice were removed using razors in ice and then stored frozen in liquid nitrogen. Colonic contents and feces were collected, weighed, and re-suspended in PBS, and their serial dilutions were then plated onto LB agar plates containing nalidixic acid. After 24 h, *C. rodentium* colonies were counted, and whether the colonies were of *C. rodentium* was confirmed using PCR with *C. rodentium*-specific primers (Bhinder et al., 2013).

### Histopathological Analysis

Excised colonic mucosal tissue specimens were fixed in 10% formalin, embedded in paraffin, and cut into 3-μm sections. The sections were then stained with hematoxylin and eosin (HE) for visualization under a microscope (100× magnification). Blind histological scoring was performed by a pathologist using a six-grade system as described by Varshney et al. (2013).

### Detection of Inflammatory Cytokines

The colonic mucosal tissues were pulverized using surgical scissors and homogenized in ice-cold PBS. The tissue homogenates were then centrifuged at 1,900× *g* at 4°C for 15 min to obtain the supernatant. The levels of pro-inflammatory cytokines IL-17, IL-6, TNF-α, and IL-10 were measured using commercial ELISA kits (eBioscience) following the manufacturer’s instructions.

### DNA Extraction and Sequence Analysis

Immediately after collection, the colonic contents were frozen, their genomic DNA was extracted, and the DNA was amplified using primers specific to the V3-V4 region of 16S rRNA gene barcodes. The samples were combined and subjected to sequencing on the Illumina MiSeq platform in accordance with the manufacturer’s instructions (Fadrosh et al., 2014). In addition, quality filtering, chimera removal, and *de novo* operational taxonomic unit (OTU) clustering were conducted using the UPARSE pipeline (Edgar, 2013). Readings were replicated, organized, and grouped into candidate OUTs, and chimeric OUTs were removed.

The taxonomic assignment of the OUTs was annotated using RDP reference (version 16) with an identity threshold of 97% in the UPARSE pipeline. The OTU table with taxonomic assignments was converted into the “biom” format for compatibility with the QIIME software (Navas-Molina et al., 2013). Alpha diversity was calculated using QIIME, for which the existing significant difference between case/control was calculated with 999 Monte Carlo permutation and Bonferroni multiple correction.

### Statistical Analysis

Data are presented as the standard error of the mean. One-way analysis of variance was used for comparison between multiple experimental groups, and the significance of the differences between the groups was determined using Duncan’s multiple range test. The sample sizes were measured to ensure statistical validity, and *p* < 0.05 was considered statistically significant.

## Results

**Figure 1:**
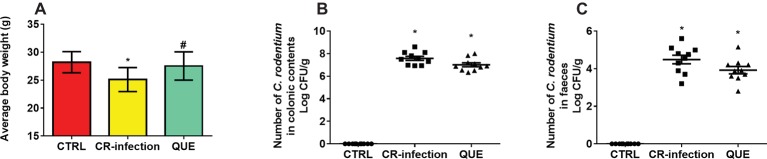
*C. rodentium* load in the colonic contents and feces of infected C57BL/6 mice on day 7 postinfection. Colonic contents and feces were collected, homogenized, serially diluted, and plated on LB agar (*n* = 10 in each group). **(A)** average body weight, numberof *C. rodentium* in **(B)** colonic contents and **(C)** faeces. To ensure that only *C. rodentium* colonies were counted, the strain in each colony was confirmed using PCR with *C. rodentium*-specific primers. * indicates *p* < 0.05 compared with the CTRL group. # indicates *p* < 0.05 compared with the CR-infection group.

**Figure 2:**
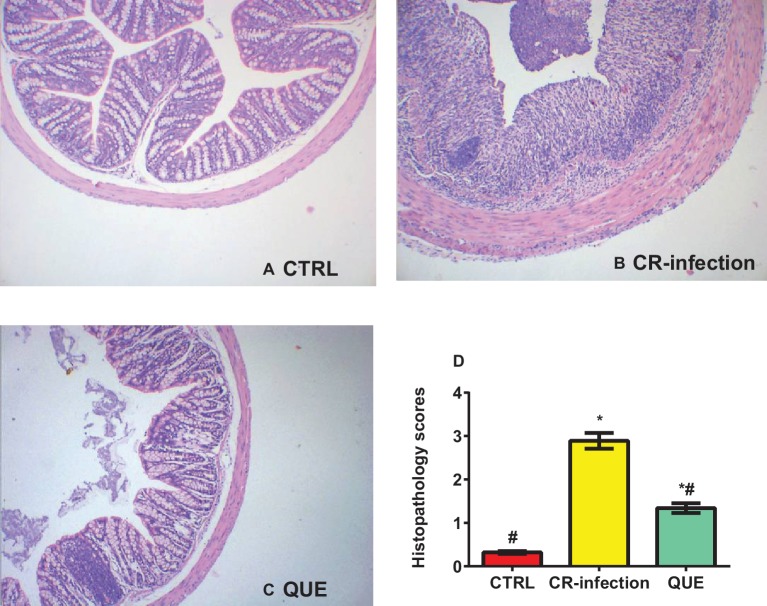
Histopathological evaluation scores for the mice on day 7 postinfection (*n* = 10 in each group). **(A)** Histological morphology of the colon (HE, ×100) in the **(A)** CTRL, **(B)** CR-infection, and **(C)** QUE groups. **(D)** Histological scores of the colon (*n* = 10 in each group). * indicates *p* < 0.05 compared with the CTRL group. # indicates *p* < 0.05 compared with the CR-infection group.

The body weights of the mice in the CTRL and QUE groups were not significantly different before and after the experiment, whereas those of the mice in the CR-infection group were lower after the experiment (Figure 1A). On day 7 postinfection, the *C. rodentium* count in the colonic contents or feces was not different between the CR-infection and QUE groups (Figures 1B,C). While comparing with the mice in the CTRL and QUE groups, the mice in the CR-infection group showed signs of colitis (Figures 2A–D).

**Figure 3:**
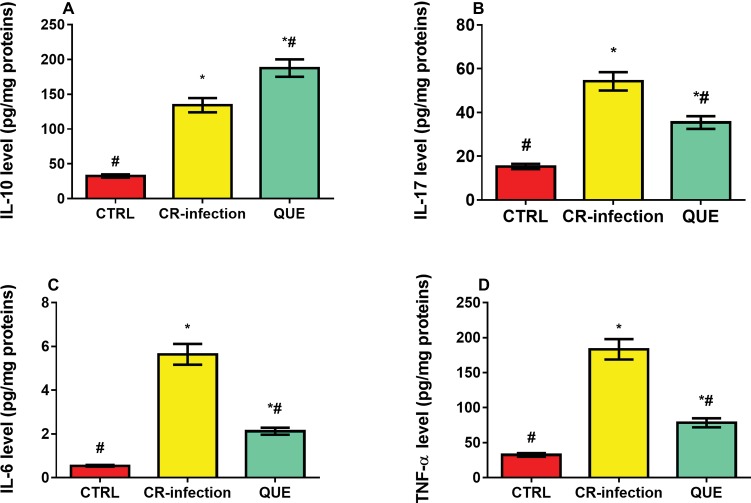
Effects of quercetin on the levels of pro-inflammatory cytokines **(A)** IL-10, **(B)** IL-17, **(C)** IL-6, and **(D)** TNF-α in mice (*n* = 10 in each group). * indicates *p* < 0.05 compared with the CTRL group. # indicates *p* < 0.05 compared with the CR-infection group.

Compared with the mice in the CTRL group, those in the CR-infection and QUE groups showed significantly elevated IL-10 (Figure 3A), IL-17 (Figure 3B), IL-6 (Figure 3C), and TNF-α (Figure 3D) levels (all *p* < 0.05). Quercetin supplementation also significantly increased the IL-10 level in the QUE mice (Figure 3A) compared with the level found in the CR-infected mice (*p* < 0.05). However, quercetin supplementation in the QUE group appeared to mitigate the *C. rodentium*-induced increases in the IL-17 (Figure 3B), IL-6 (Figure 3C), and TNF-α (Figure 3D) levels (*p* < 0.05).

**Figure 4:**
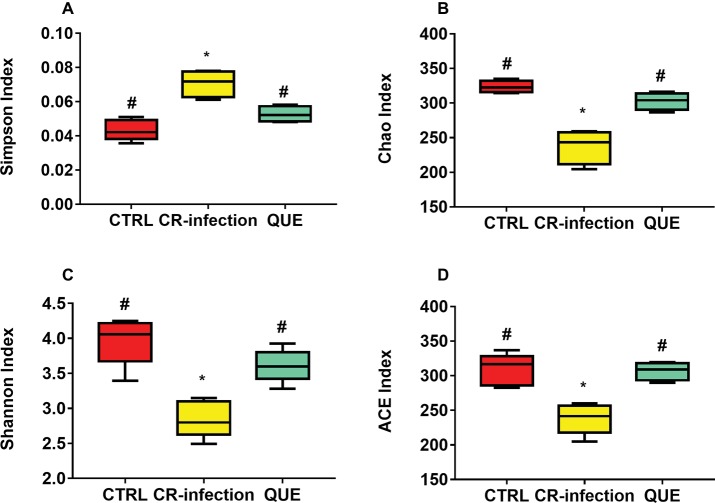
Diversity indices of bacterial communities in the mouse colonic contents (*n* = 10 in each group). Box plots show the differences in the microbiome diversity between the CTRL, CR-infection and QUE groups in terms of the **(A)** Simpson, **(B)** chao1, **(C)** Shannon, and **(D)** ACE indices. * indicates *p* < 0.05 compared with the CTRL group. # indicates *p* < 0.05 compared with the CR-infection group.

Amplification of the V3-V4 region of the 16S rRNA gene obtained from the colonic contents of CTRL, CR-infected, and QUE groups provided raw readings (35,673, 37,894, and 33,511, respectively) to facilitate the assessment of the effects of *C. rodentium* infection and dietary quercetin on bacterial communities. Following trimming, assembly, and quality filtering, 2,895 OTUs were detected. Figure 4 presents the Shannon and Simpson diversity indices and microbial richness indices (Chao1 and ACE) in the groups. Compared with the CTRL group, the CR-infection group showed significantly increased Simpson index (*p* < 0.05) (Figure 4A) and decreased Chao1 (Figure 4B), Shannon (Figure 4C) and ACE indices (Figure 4D) (all, *p* < 0.05). Compared with the CR-infection group, the QUE group showed increased Chao1 (Figure 4B), Shannon (Figure 4C), and ACE indices (Figure 4D) (all, *p* < 0.05) and decreased Simpson index (Figure 4A) (*p* < 0.05). However, the observed alpha diversity of the microbiota showed no significant difference between the CTRL and QUE groups.

**Figure 5:**
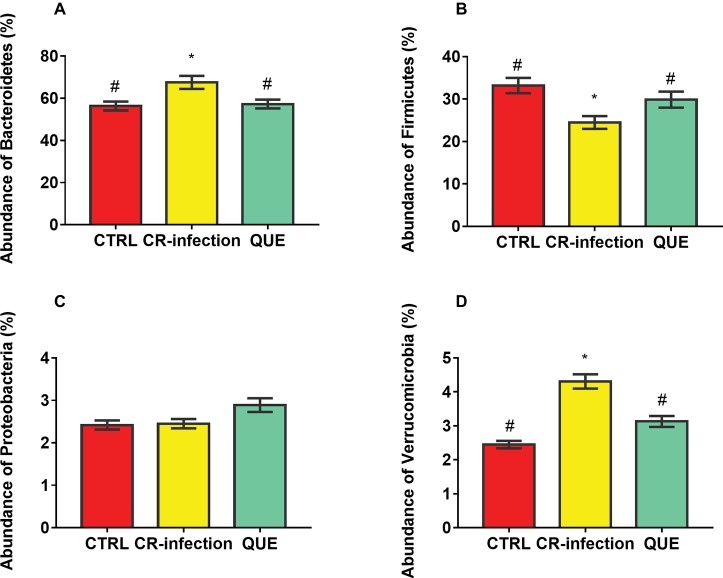
Colonic microbial composition at the phylum level. Relative abundances of **(A)** Bacteroidetes, **(B)** Firmicutes, **(C)** Proteobacteria, and **(D)** Verrucomicrobia members in the mouse colonic contents (*n* = 10 in each group). * indicates *p* < 0.05 compared with the CTRL group. # indicates *p* < 0.05 compared with the CR-infection group.

Taxon-dependent analysis was used to determine the intestinal microbiota taxonomy, and Bacteroidetes, Firmicutes, Proteobacteria, and Verrucomicrobia were found to be the most abundant phyla (Figures 5A–D). Their relative abundances were 56.32, 33.18, 2.42 and 2.45%, respectively, in the CTRL group; 67.54, 24.47, 2.45, and 4.31%, respectively, in the CR-infection group; and 57.24, 29.87, 2.89, and 3.13%, respectively, in the QUE group.

**Figure 6:**
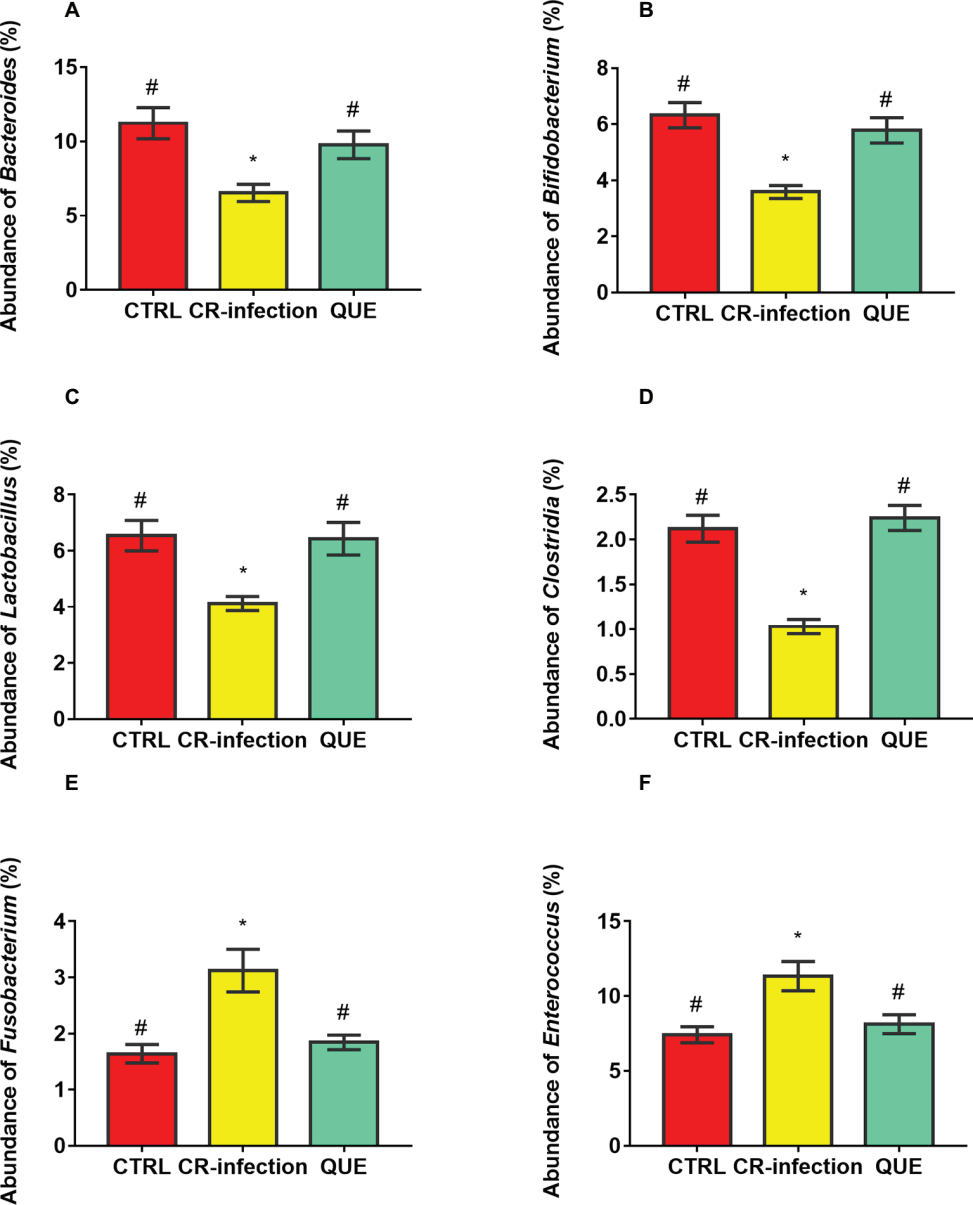
Colonic microbial composition at the genus level. Relative abundances of **(A)** *Bacteroides*, **(B)** *Bifidobacterium*, **(C)** *Lactobacillus*, **(D)** *Clostridia*, **(E)** *Fusobacterium*, and **(F)** members in the colonic contents (*n* = 10 in each group). * indicates *p* < 0.05 compared with the CTRL group. # indicates *p* < 0.05 compared with the CR-infection group.

Microbial populations at the genus level in the colonic contents were also investigated (Figures 6A–F). The populations of *Bacteroides*, *Bifidobacterium*, *Lactobacillus*, and *Clostridia* were decreased (*p* < 0.05), and those of *Fusobacterium* and *Enterococcus* were increased in the CR-infection group (*p* < 0.05) compared with those in3 the CTRL group. Notably, compared with the CR-infection group, the QUE group showed enhanced populations of *Bacteroides*, *Bifidobacterium*, *Lactobacillus*, and *Clostridia* but suppressed populations of *Fusobacterium* and *Enterococcus* (*p* < 0.05) because of quercetin supplementation.

## Discussion

Because of their inherent susceptibility to EPEC or EHEC, mice have been the most frequently used model for studying *C. rodentium*-induced intestinal infection or intestinal EPEC or EHEC infection. Although *C. rodentium* rarely causes intestinal diseases in humans, it can colonize all mice strains, causing either fatal or virtually asymptomatic illness depending on the mouse strain (Coleman et al., 2014; Scholz et al., 2016). The CD-1 and C57BL/6 mouse strains develop only subclinical symptoms and are considered resistant to *C. rodentium*-induced colitis, whereas FVB/N and C3H/HeJ mice strains exhibit *C. rodentium* infection and are considered susceptible (Borenshtein et al., 2008). In particular, mouse models of *C. rodentium*-induced infection are the most useful for studying infectious diseases and colitis in mice because they have been well documented for host responses to pathogenic bacteria. Our previous study showed that quercetin or quercetin monoglycoside supplementation can prevent dextran sulphate sodium-induced colitis (Hong and Piao, 2018). In that study, mice were fed quercetin to protect the gut and allow rapid recovery after *C. rodentium* infection. The results indicated that quercetin supplementation provided therapeutic benefits in the *C. rodentium*-induced infection model of gastrointestinal injury. Inflammatory responses and intestinal microflora composition were the most important determinants of host susceptibility.

Dietary preference has a major impact on the gut microbial composition throughout human life (Conlon and Bird, 2014; Yin et al., 2018). IBD has been shown to be associated with alterations in the human gut microbial composition (Willing et al., 2010; Tong et al., 2013). Decreased microbiome diversity has been observed in CD patients (Ma et al., 2018) and in monozygotic discordant twins with CD (Dicksved et al., 2008). Decreased microbiome diversity has mainly been attributed to reduced diversity of Firmicutes members and has been linked to temporal instability in both CD and UC (Coleman et al., 2014). Decreased diversity has also been observed in inflamed and noninflamed tissues, and CD patients generally exhibit reduced bacterial loads at inflammation sites (Willing et al., 2010; Zhu et al., 2018).

Dysregulation of the mucosal immune system can cause IBD and a pathogenic immune response against gut flora (Xu et al., 2014; Haag and Siegmund, 2015). The present study suggests that quercetin affects the progress of microbiota-associated diseases. Notably, quercetin supplementation increased gut microbial diversity, which may improve gut protection. In IBD patients, gut microbiota dysbiosis is a common occurrence, typically manifesting as a superfluity of facultative anaerobic bacteria and a simultaneous deficiency of obligate anaerobic bacteria of the classes Bacteroidia and Clostridia (Minamoto et al., 2015; Stecher, 2015). According to Wu et al., long-term dietary patterns may affect the ratios of Bacteroides, Firmicutes, and Prevotella populations, whereas short-term dietary changes may show limited effects (Wu et al., 2011). In addition, Zimmer et al. stated that strict vegan or vegetarian diets significantly decrease *Bacteroides*, Enterobacteriaceae, and *Bifidobacterium* populations (Zimmer et al., 2012). Enterobacteriaceae populations have been consistently found to be elevated in IBD patients. Therefore, further studies are warranted to evaluate the effects of both long-term and short-term dietary changes on gut microbiota and consequently on IBD (Azad et al., 2018; Guan and Lan, 2018).

Inflammation can also considerably affect the gut microbiota. Severe inflammation has been reported to increase the relative abundance of *Salmonella* and similar pathogens (Varshney et al., 2013; Saltzman et al., 2018). In the present study, *C. rodentium*-infected mice showed significantly increased levels of proinflammatory cytokines IL-6, TNF-α, and IL-17 in the colonic mucosal tissues compared with the CTRL mice (not infected). It has been demonstrated that quercetin protects against colonic damage linked to increases in the TNF-α level (Donder et al., 2018; Ju et al., 2018). In the present study, quercetin supplementation reduced localized production of inflammatory cytokines, which in turn promoted alterations in bacterial flora composition associated with rapid repair. This finding indicates that quercetin might restore the appropriate host-microbe relationship required to manage colitis by restoring the proinflammatory, anti-inflammatory, and bactericidal functions of enteric macrophages (Ju et al., 2018).

In summary, the findings of this study on the *C. rodentium*-infected mouse model demonstrated that quercetin could reduce the pathological effects of *C. rodentium*. This finding suggests that dietary quercetin can directly stimulate the immune system to reduce inflammation and restore gut microbial balance. Future studies using human subjects are desirable to confirm these effects of quercetin on inflammatory markers and provide a more comprehensive understanding of the quercetin-induced variations in the human gut microbiota.

## Data Availability

All the data are available upon reasonable request at Dr. Rui Lin, pubmed1128@126.com.

## Ethics Statement

The current research was conducted according to Chinese animal welfare guidelines and following the granting of approval by the Animal Care and Use Committee of General Hospital of the Tianjin Medical University.

## Author Contributions

RL designed the experiment. RL, MP, and YS performed the experiment and statistical analysis. RL finished the draft of the manuscript. MP and YS revised the manuscript. All the authors read and approved the manuscript.

### Conflict of Interest Statement

The authors declare that the research was conducted in the absence of any commercial or financial relationships that could be construed as a potential conflict of interest.
